# Why do you need a biostatistician?

**DOI:** 10.1186/s12874-020-0916-4

**Published:** 2020-02-05

**Authors:** Antonia Zapf, Geraldine Rauch, Meinhard Kieser

**Affiliations:** 1 Department of Medical Biometry and Epidemiology, University Medical Center Hamburg-Eppendorf, Martinistr. 52, 20246 Hamburg, Germany; 2 Charité - Universitätsmedizin Berlin, corporate member of Freie Universität Berlin, Humboldt-Universität zu Berlin, and Berlin Institute of Health, Institute of Biometry and Clinical Epidemiology, Charitéplatz 1, 10117 Berlin, Germany; 3 Institute of Medical Biometry and Informatics, Heidelberg University Hospital, Im Neuenheimer Feld 130.3, 69120 Heidelberg, Germany

**Keywords:** Medical research, Biostatistician, Tasks, Responsibilities

## Abstract

The quality of medical research importantly depends, among other aspects, on a valid statistical planning of the study, analysis of the data, and reporting of the results, which is usually guaranteed by a biostatistician. However, there are several related professions next to the biostatistician, for example epidemiologists, medical informaticians and bioinformaticians. For medical experts, it is often not clear what the differences between these professions are and how the specific role of a biostatistician can be described. For physicians involved in medical research, this is problematic because false expectations often lead to frustration on both sides. Therefore, the aim of this article is to outline the tasks and responsibilities of biostatisticians in clinical trials as well as in other fields of application in medical research.

## Background

What is a biostatistician, what does he or she actually do and what distinguishes him or her from, for example, an epidemiologist? If we would ask this our main cooperation partners like physicians or biologists, they probably could not give a satisfying answer. This is problematic because false expectations often lead to frustration on both sides. Therefore, in this article we want to clarify the tasks and responsibilities of biostatisticians.

There are some expressions which are often used interchangeably to the term ‘biostatistician’. In here, we will use the expression ‘(medical) biostatistics’ as a synonym for ‘medical biometry’ and ‘medical statistics’, and analogously we will do for the term ‘biostatistician’.

In contrast to the clearly defined educational and professional career steps of a physician, there is no unique way of becoming a biostatistician. Only very few universities do indeed offer studies in biometry, which is why most people working as biostatisticians studied something related, subjects such as mathematics or statistics, or application subjects such as medicine, psychology, or biology. So a biostatistician cannot be defined by his or her education, but must be defined by his or her expertise and competencies [1]. This corresponds to our definition of a biostatistician in this article. The International Biometric Society (IBS) provides a definition of biometrics as a ‘field of development of statistical and mathematical methods applicable in the biological sciences’ [2]. In here, we will focus on (human) medicine as area of application, but the results can be easily transferred to the other biological sciences like, for example, agriculture or ecology. As mentioned above, there are some professions neighbouring biostatistics, and for many cooperation partners, the differences between biostatisticians, medical informaticians, bioinformaticians, and epidemiologists are not clear. According to the current representatives of these four disciplines within the German Association for Medical Informatics, Biometry and Epidemiology (GMDS) e. V.:

- ‘Medical biostatistics develops, implements, and uses statistical and mathematical methods to allow for a gain of knowledge from medical data.’ ‘Results are made accessible for the individual medical disciplines and for the public by statistically valid interpretations and suitable presentations’ (authors’ translation from [3]).
- ‘Medical informatics is the science of the systematic development, management, storage, processing, and provision of data, information and knowledge in medicine and healthcare’ (authors’ translation from [4]).
- Bioinformatics is a science for ‘the research, development and application of computer-based methods used to answer biomolecular and biomedical research questions. Bioinformatics mainly focusses on models and algorithms for data on the molecular and cell-biological level’ [5].
- ‘Epidemiology deals with the spread and the course of diseases and the underlying factors in the public. Apart from conducting research into the causes of disease, epidemiology also investigates options of prevention’ (authors’ translation from [6]).

Another discipline is data science, which is a relatively new expression used in a multitude of different contexts. Often it is meant as a global summarizing term covering all of the above mentioned fields. As there is no common agreement on what data science is and as this term does not correspond to a uniquely defined profession, this expression will not be discussed in more detail.

The self-descriptions as stated above are rather general and not necessarily complete. Therefore, we will in the following describe the specific tasks and responsibilities of biostatisticians in different important application fields in more detail. This allows us to specify what cooperation partners may (or may not) expect from a biostatistician. Furthermore, clarification of the roles of all involved parties and their successful implementation in practice will overall lead to more efficient collaborations and higher quality.

## Main text

### Tasks and responsibilities of biostatisticians

There are many medical areas where biostatisticians can contribute to the general research progress. These fields of application and the related biostatistical methods are not strictly separated, but there are many overlaps and a classification of the related methodology can be done in various ways. We consider in the following the important application fields of clinical trials, systematic reviews and meta-analysis, observational and complex interventional studies, and statistical genetics to highlight the tasks and responsibilities of biostatisticians working in these areas.

### Biostatisticians working in the area of clinical trials

The tasks of biostatisticians in clinical trials are not limited to the analysis of the data, but there are many more responsibilities. It is a quite misguided view that biostatisticians are only required after the data has been collected. According to Lewis et al. (1996), statistical considerations are not only relevant for the analysis of data but also for the design of the trial [7]. This is not a personal view, but general consensus. It is demanded by the ethics committee and confirmed by the principle investigator and / or the sponsor when stating that the clinical trial will be conducted according to Good Clinical Practice (GCP). The corresponding guideline E6 from the *International Council for Harmonisation of Technical Requirements for Pharmaceuticals for Human Use* (ICH) explicitly states that statistical expertise should be utilized throughout all stages [8]. In there, it is stated in Section 5.4.1: ‘The sponsor should utilize qualified individuals (e.g. biostatisticians, clinical pharmacologists, and physicians) as appropriate, throughout all stages of the trial process, from designing the protocol and CRFs [case report forms, AZ] and planning the analyses to analyzing and preparing interim and final clinical trial reports.’ Mansmann et al. [9] provided a more specific guidance about good biometrical practice in medical research and the responsibilities of a biostatistician. In there, the responsibility of a biostatistician is described as a person participating in the planning and the execution of a study, in the dissemination of the results and in statistical refereeing. These are very general descriptions of the tasks and responsibilities of biostatisticians. In the following, we will explain the biostatistician’s mission in more detail based on the guidance on good biometrical practice [9] and on the E9 guideline from the ICH about Statistical Principles for Clinical Trials [10].

In the initial phase of a medical research project, a biostatistician should actively participate in the assessment of the relevance and the feasibility of the study. During the planning phase, the biostatistician should already be involved in the discussion of general study aspects as outlined in more detail below. It is evident that the physician must provide the framework for this. However, the biostatistician can and should point out important biostatistical issues which will have important influence on the whole construct of the study. Therefore, an important part of the biostatistician’s work is to be done long before a study can start. For example, the appropriate study population (special subgroups or healthy subjects in early phases versus large representative samples of the targeted patient population in confirmatory trials) and reasonable primary and secondary endpoints (e.g. suitable to the study aim, objectively measurable, clearly and uniquely defined) need to be identified. He also should make the physician aware of the potential problems with multiple or composite primary endpoints and with surrogate or categorised (especially dichotomized) variables. Another very important topic related to the general study design is blinding and randomisation as techniques to avoid bias. Moreover, the comparators or treatment arms must be specified and it has to be defined how they are embedded in the general study design (for example parallel or crossover). It also has to be specified the aim in whether is to show superiority or non-inferiority of the new treatment and whether interim analyses are reasonable (group sequential designs). Moreover the procedures for data capture and processing have to be discussed at this point. Only after fixing all these planning aspects, the biostatistician can provide an elaborated sample size calculation.

During the ongoing study, main tasks and responsibilities consist of biostatistical monitoring (for example as part of a data safety monitoring board) and performing interim analyses (if planned). If any modifications of the study design are urgently required during the ongoing trial (for example changes within an adaptive designs, or early stopping after an interim analysis), the biostatistician has to be involved in the discussions and decisions as otherwise the integrity of the study can be damaged.

The main data analysis is performed after all patients were recruited and fully observed. However, the statistical methods applied within the data analysis must already be specified during the planning phase within the study protocol. The study protocol should already be as detailed as possible in particular with regard to the analysis of the primary endpoint(s). In addition, the statistical analysis plan (SAP), which must be finalized before start for the data analysis, provides a document which describes all details on the primary, secondary and safety analyses. It also covers possible data transformations, applied point and interval estimators, statistical tests, subgroup analyses, and the consideration of interactions and covariates. Furthermore, the used data sets (for example intention to treat or per protocol), the handling of missing values, and a possible adjustment for multiplicity should be described and discussed. Another important issue is how the integrity of the data and the validity of the statistical software can be guaranteed.

In a last step, after the finalization of the data analysis according to the SAP, the biostatistician contributes to reporting the results in the study report as well as in the related publications submitted to medical journals. He or she is responsible for the appropriate presentation and the correct interpretation of the results.

To sum up, in clinical studies, the tasks and responsibilities of biostatisticians thus extend from the planning phase, through the execution of the study to data analysis and publication of the results. In particular, a careful study planning, in which the contribution of a biostatistician is indispensable, is essential to obtain valid study results.

### Biostatisticians working in the area of systematic reviews and meta-analysis

To judge the level of evidence of medical research, different systems of evidence grading were suggested. The recent grading system from the Oxford Centre for Evidence-Based Medicine (OCEBM) defines ten evidence levels. The highest level is a systematic review of high quality studies for the therapeutic as well as for the diagnostic and prognostic context [11]. The need for such reviews results from the huge amount of articles in the medical literature, which has to be aggregated appropriately [12]. As Gopalakrishnan and Ganeshkumar describe, the aim of a systematic review is to ‘systematically search, critically appraise, and synthesize on a specific issue’ [13]. A meta-analysis, which additionally provides a quantitative summary, can be part of a systematic review, if a reasonable number of individual studies are available. The task and responsibilities of biostatisticians in this field are described in the following. As in clinical trials, the biostatistician should already be involved during the planning phase of a systematic review/meta-analysis to discuss the design aspects and the feasibility. Beside the literature search and the collection of the study data (most often not available on an individual patient level), the assessment of the study quality and the risk of bias are important topics. There are different tools for the assessment, like the GRADE approach (Grading of Recommendations, Assessment, Development and Evaluation) [14] or the QUADAS-2 tool (Quality Assessment of Diagnostic Accuracy Studies) for diagnostic meta-analyses [15]. A general description of these approaches can be found in the Cochrane Handbook [16]. The main task of biostatisticians in the field of systematic reviews is then to perform the meta-analysis itself including the calculation of weighted summary measures, creation of graphs, and performing subgroup and sensitivity analyses. As a last step, the biostatistician should again support the physicians in interpreting und publishing the results.

In summary, the tasks and responsibilities of biostatisticians in the field of systematic reviews and meta-analyses relate to the proper planning, the evaluation of the quality of the individual studies, the meta-analysis itself and the publication of the results.

### Biostatisticians working in the area of observational and complex interventional studies

In observational studies, where confounding plays a major role, statistical modelling aims at incorporating, investigating, and exploiting relationships between variables using mathematical equations. Other important examples for application of the related techniques are longitudinal data measured repeatedly in time for the same subject or data with an inherent hierarchical structure, for example data of patients observed in different departments within various clinics. Valid conclusions from the analysis are only obtained if the functional relationship between the variables is correctly taken into account [17]. Another prominent task of statistical modelling is prediction, for example to forecast a future outcome of patients. Frequently, the relationship between the involved variables is complex. For example, patients may undergo several states between start of observation and outcome and the transitions between these states as well as potential competing risks have to be adequately considered (see, for example, Hansen et al. [18]). Extrapolation is another field of growing interest where techniques of statistical modelling are indispensable. This process can be defined as ‘extending information and conclusions available from studies in one or more subgroups of the patient population (source population), or in related conditions or with related medicinal products, to make inferences for another subgroup of the population (target population), or condition or product’ [19]. For example, clinical trial data for adults may be used to assist the development of treatments for children [20]. Last but not least, statistical modelling may be of help in situations where data of different origin shall be synthesized to increase evidence, for example, from randomized clinical trials, observational studies, and registries. These examples are by far not exhaustive and illustrate the wide spectrum of potential data sources and applications. It is obvious that there are direct connections to the two working areas of biostatisticians described in the preceding subsections, and consequently there are substantial overlaps in the related tasks and responsibilities. As in the other working areas considered, the biostatistician is responsible for choosing a correct and efficient analysis method that includes all relevant information. Due to the complexity of statistical models, this point is especially challenging here. Furthermore, it is the task of biostatisticians to decide whether the mandatory data required to adequately map the underlying relationships are included in the available data set, whether data quality and completeness is sufficiently high to justify a reliable analysis, and to define appropriate methods dealing with missing values. It is highly recommended to prepare an SAP not only for clinical trials (see Biostatisticians working in the area of clinical trials section) but also for analyses using methods of statistical modelling.

Again, the biostatistician is responsible not only for a proper planning and conducting of the analyses but also for appropriate interpretation and presentation of the results. The particular challenge for biostatisticians in this area is to choose appropriate statistical models for the analysis of data with a complex structure.

### Biostatisticians working in the area of statistical genetics

Biostatisticians working in the fields of genetics and genomics are often the responsible persons for the final integration of multidisciplinary expertise in mathematics, statistics, genetics, epidemiology, and bioinformatics to only cite some common ingredients. Planning tasks include the design of research studies, which may pursue exploratory and/or confirmatory objectives. There exist a broad range of possible study designs which make use of well-differentiated modelling techniques. Generated data are often pre-processed by bioinformaticians before it reaches the biostatistician. Pre-processing of sequencing data, for instance, usually comprises quality control of sequenced reads, alignment to the human reference genome and markup of duplicates previously to the identification of somatic mutations and indels. Good knowledge of the limitations of applied pre-processing techniques by the statistician is often very helpful. A strong background and a deep understanding of genetics and genomics as well as an interdisciplinary thinking are a must for biostatisticians working in this area. These competences will be even more important in future. For example, emerging fields of research like Mendelian randomization where genetic variants are used as instruments to predict causality will require an even stronger interaction between statistics and genetics.

In the field of statistical genetics, tasks and responsibilities relate in particular to study planning, critical review of pre-processing, and data analysis using appropriate statistical models.

## Discussion

Biostatistics mainly addresses the development, implementation, and application of statistical methods in the field of medical research [3]. Therefore, an understanding of the medical background and the clinical context of the research problem they are working on is essential for biostatisticians [21]. Furthermore, a specific professional expertise is inevitable, and also soft skill competencies are very important. Regarding the professional expertise, the ICH E9 guideline states that a trial statistician should be qualified and experienced [10]. Qualification, which means biostatistical expertise, covers methodological background (mathematics, statistics, and biostatistics), biostatistical application, medical background, medical documentation, and statistical programming. The experience relates to consulting, planning, conducting and analysing medical studies. Jaki et al. [22] gave a review of training provided by existing medical statistics programmes and made recommendations for a curriculum for biostatisticians working in drug development. Regarding the soft skills of a biostatistician, some literature exists (for example [23] or [24]). Furthermore, Zapf et al. [1] summarize the professional expertise and the needed soft skills of a biostatistician according to the CanMEDS framework [25], which was developed to describe the required abilities of physician (the original abbreviation ‘Canadian Medical Education Directions for Specialists’ is no longer in use).

In this article, we did not explicitly consider the recently upcoming field of biomedical data science which is applied in many different areas of medical research such as, for example, individualized medicine, omics research, big data analysis. The tasks and responsibilities of biostatisticians working in this domain are not different from those reported above but in fact include all mentioned aspects [26].

## Conclusion

There is evidently an overlap between the tasks and responsibilities of medical biostatisticians and neighbouring professions. However, all disciplines have different focuses. Important application fields of biostatistics are clinical studies, systematic reviews / meta-analysis, observational and complex interventional studies, and statistical genetics.

In all fields of biostatistical activities, the working environment is diverse and multi-disciplinary. Therefore, it is essential for fruitful, efficient, and high-quality collaborations to clearly define the tasks and responsibilities of the cooperating partners. In summary, the tasks and responsibilities of a biostatistician across all application areas cover active participation in a proper planning, consultation during the entire study duration, data analysis using appropriate statistical methods as well as interpretation and suitable presentation of the results in reports and publications. These tasks are similarly formulated by the ICH E6 guideline concerning good clinical practice [8].

## Data Availability

Not applicable.

## Abbreviations

CanMEDS: Canadian Medical Education Directions for Specialists
CRF: Case report form
GCP: Good Clinical Practice
GMDS: German Association for Medical Informatics, Biometry and Epidemiology
GRADE: Grading of Recommendations, Assessment, Development and Evaluation
IBS: International Biometric Society
ICH: International Council for Harmonisation of Technical Requirements for Pharmaceuticals for Human Use
OCEBM: Oxford Centre for Evidence-Based Medicine
QUADAS: Quality Assessment of Diagnostic Accuracy Studies
SAP: Statistical analysis plan
